# Local Anesthesia by Eucaine in Rectal Diseases

**Published:** 1907-04

**Authors:** 


### Local Anesthesia by Eucaine in Rectal Diseases

Charles B. Kelsey, Surgeon at the Post-Graduate Hospital, New York, in a paper on "The Office Treatment of Diseases of the Rectum, with a Description of Some New Methods," (*Medical Record,* October 21, 1905,) recommends the use of local anesthesia in rectal diseases. When eucaine became known, cocaine was abandoned, the former having equal effect with much less constitutional disturbance.

The recent exploiting of simple water as a local anesthetic has not appealed to him. It is certainly not new, for it has been a scientific curiosity for years; but the practical advantage of injecting an ounce of water into the tissues to secure an effect that can as well be gained by ten drops of a very weak and absolutely safe solution of eucaine is not manifest, while the disadvantages are obvious. Moreover, eucaine in many cases can be efficiently applied on a piece of cotton allowed to rest on the spot and need not be injected into the tissues at all to produce full anesthesia.

Thus, he bathes fissure in strong eucaine, or injects a weaker eucaine under it, till all sensation is abolished, and incises freely. Eucaine anesthesia is equally useful in cases of pruritus ani and of fistula, as well as in hemorrhoids, where he does galvano cautery.

He found a distinct advantage in using eucaine instead of general anesthesia, especially in old or very nervous and timid patients, in cases where the tumors can easily be brought outside the anus and the surgeon's work is all before him.

